# Prevalence, incidence and remission of urinary incontinence in women: longitudinal data from the Norwegian HUNT study (EPINCONT)

**DOI:** 10.1186/1471-2490-13-27

**Published:** 2013-05-30

**Authors:** Marit Helen Ebbesen, Steinar Hunskaar, Guri Rortveit, Yngvild Skaatun Hannestad

**Affiliations:** 1 Research Group for General Practice, Department of Global Public Health and Primary Health Care, University of Bergen, Kalfarveien 31, Pb 7804, 5020 Bergen, Norway; 2 Uni Health, Uni Research, Kalfarveien 31, Pb 7810, Bergen, 5020, Norway; 3 Research Unit for General Practice, Uni Health, Uni Research, Bergen, Norway

**Keywords:** Prevalence, Incidence, Remission, Urinary incontinence, Diabetes, Women, Epidemiology, EPINCONT, HUNT

## Abstract

**Background:**

To determine incidence and remission of UI as well as changes in UI prevalence in the Norwegian EPINCONT surveys.

**Methods:**

The EPINCONT surveys were conducted in the county of Nord-Trøndelag, Norway, as part of two large cross-sectional health surveys (HUNT2 and HUNT3) in 1995 – 1997 (EPINCONT1 (E1)), and 2006 – 2008 (EPINCONT2 (E2)). EPINCONT collected information about prevalence of UI, as well as information about type and severity of UI.

**Results:**

A 16% relative increase in UI prevalence was found in 11 years. The women who answered E2 were significantly older, had a higher BMI and higher prevalence of diseases such as asthma, diabetes and angina compared with the women who answered E1.

The incidence of UI was 18.7%. Increase in BMI (OR 1.03, 95% CI: 1.02 – 1.04), weight increase (OR 1.29 (95% CI: 1.14 – 1.45) for gaining 3 – 10 kilos and OR 1.71 (95% CI: 1.47 – 1.99) for gaining 10 kilos or more) and parity (OR 1.37 (95% CI: 1.04 – 1.79) for 1 childbirth, OR 1.28 (95% CI: 1.03 – 1.61) for 2 childbirths, and OR 1.56 (95% CI: 1.26 – 1.95) for 3 or more childbirths when participating in E2) were all found to be associated with increased odds of incident UI in adjusted regression analyses. Increasing age reduced the odds of incident UI. The 11 year remission of UI was 34.1%. Increasing age (OR 0.98, 95% CI: 0.98 – 0.99), increasing BMI (OR 0.96, 95% CI: 0.95 – 0.98) and large weight gains of 10 kilos or more (OR 0.69, 95% CI: 0.54 – 0.88) were all associated with reduced remission of UI.

**Conclusion:**

Crude UI prevalence increased between the studies. Changes in known risk factors for UI such as age, BMI, weight and parity could explain some of the relative increase in prevalence, and were also found to be associated with either incidence of UI, remission of UI or both.

## Background

There are abundant studies on prevalence of urinary incontinence (UI), and a large prevalence span has been reported [1]. Few cohort studies have reported change in prevalence [2-4], and repeated cross- sectional studies based on entire adult female populations (20+) are to the best of our knowledge scarce [5]. Studies reporting incidence of UI are increasing in number, and a recent review of studies on overactive bladder and UI found annual incidence rates between 0.9 and 19% [6]. Differences in study design [7-9], definitions used [2,10] and interval between studies [3,11] could possibly explain some of the large variation.

The Norwegian HUNT study (The Nord-Trøndelag Health Study) can provide useful information about changes in UI prevalence as well as incidence and remission of UI in large adult female populations, since all women aged 20 years or older in a Norwegian county were invited to participate at two different points in time (1995 - 1997 and 2006 - 2008). The UI part of HUNT, the EPINCONT substudy (Epidemiology of Incontinence in the County of Nord-Trøndelag) was included on both occasions. The EPINCONT surveys use validated questions to determine severity of UI [12,13] as well as definitions of UI in concordance with the recent recommendations from the International Continence Society (ICS) [14]. As EPINCONT is part of a large prospective general health study extensive information about other health and life style topics is provided. This provides opportunities for assessing predictors for change in UI prevalence, incidence and remission of UI, as well as clarifying the roles of known or less determined risk factors for UI, such as diabetes.

The current article presents prevalence as well as incidence and remission data on UI in the Norwegian EPINCONT population, with two data points approximately 11 years apart. We also investigate associations with some known risk factors for UI such as age, BMI, parity and diabetes, and discuss their possible influence on the changes found.

## Methods

The Norwegian HUNT study was performed in the county of Nord-Trøndelag at three different occasions, baseline in 1984 - 1986 (HUNT1), and two follow up surveys in 1995 -1997 (HUNT2, baseline for EPINCONT) and 2006 - 2008 (HUNT3). The incontinence part (EPINCONT) was first introduced in the HUNT2 survey (1995 - 1997, EPINCONT1 (E1)), with follow up questions in HUNT3 (2006 - 2008, EPINCONT2 (E2)). Everyone living in the county aged 20 years or older was invited by mail to participate. Along with the invitation was Questionnaire 1 (Q1). Screening stations were set up on different locations in the county, and those who chose to participate were to bring Q1 with them to one of these screening stations. At the screening station the participants underwent some clinical examinations, and additional information was gathered by interviewing the participants as well as providing everyone with Questionnaire 2 (Q2), which should be filled in at home and then returned by mail in a prepaid envelope. The EPINCONT part was included in Q2. Those answering affirmative to some specific questions in Q1 also received Questionnaire 3 (Q3) to collect depth information on that specific topic, e.g. diabetes. Some of the information collected by questionnaires in HUNT2 was collected by interviews at the screening stations in HUNT3, e.g. parity.

The EPINCONT part consisted of the same questions on both occasions. The women first answered yes or no to the entry question “Do you have involuntary loss of urine”? Severity of UI was then determined by asking the women about amount (3 levels) and frequency (4 levels) of leakage. Amount and frequency were multiplied and Sandvik’s Severity index calculated in order to classify the women as having slight, moderate, severe or very severe UI [13]. Due to a technical error the question about amount of leakage only contained two levels in approximately 25% of the questionnaires in the E1 survey, and hence these women are missing on the 4-level Severity index analyses. Information about type, duration, impact of UI and help seeking due to UI were also collected [15]. Women participating in E2 also answered a question about treatment of UI.

The women were defined as incontinent if they answered affirmatively to the entry question about any involuntary leakage of urine. Those who answered no to this question or had a missing answer were still included as incontinent if they provided answers to the questions about type, frequency and amount of leakage (all three had to be answered), in accordance with the definitions used in previous publications from the E1 survey [15]. Incidence of UI was defined as having no UI in E1 and having UI in E2, whilst remission of UI was defined as having UI in E1 but not in E2. Average yearly incidence and remission of UI were calculated to compare the results from EPINCONT to existing literature. Average yearly incidence/remission of UI was determined by dividing the incidence/remission by the years passed between surveys.

For the crude prevalence analyses, women answering E1 or E2 were analysed separately (Tables 1, 2 and 3), whilst in the analyses of incidence and remission of UI longitudinal data was used; hence only women who participated in both EPINCONT surveys were included in these analyses. Analyses presented in Tables 2, 3, 4 and 5 are limited to the women who had given a valid answer to the incontinence questions in one or both of the EPINCONT surveys.The women were analysed in 5 - year age groups with regards to crude prevalence of any UI as well as analyses on type and severity of UI. For analyses of incidence and remission (Table 4) the women were divided into 3 groups 20 - 39, 40 - 54, 55+ years of age (age when participating in E1 was used). Known risk factors for UI such as age, BMI and parity were adjusted for in the multivariate regression analyses. Based on previous publications [16] and preliminary analyses smoking was also included amongst the adjustment variables (Table 5). Age, BMI and smoking status were obtained from the E1 survey whilst parity was obtained from E2, hence age, BMI and smoking status could be investigated as possible risk factors for incidence and remission of UI. Parity (E2) and weightchange could have occurred after incidence/remission of UI, so it is only possible to investigate associations between these variables and incidence/remission of UI. Diabetes was investigated separately as a risk factor for incidence and remission of UI, but was not included in the final regression model.

**Table 1:** Participation rates for the HUNT and EPINCONT surveys

	**HUNT2**		**HUNT3**		**Both HUNT2 and HUNT3**	
	**N**	**%**	**N**	**%**	**N**	**%**
Women invited to HUNT	47 177	100.0	47 415	100.0	35 263	100.0
Answered Q1	34 662	73.5	27 758	58.5	20 465	58.0
Attended screening (source population EPINCONT)	34 548	73.2	27 691	58.4	20 417	57.8
Answered EPINCONT^a^	27 992	81.0	21 804	78.7	14 606	71.5

^a^: Percentage of source population.

**Table 2:** Characteristics of the women participating in EPINCONT1 (E1, 1995 – 1997), EPINCONT2 (E2, 2006 – 2008) and both EPINCONT surveys

	**E1**		**E2**		**P**^**b**^	**E1 and E2**^**c**^		**P**^**d**^
**Participants**^**a**^	**N = 27 992**		**N = 21 804**			**N = 14 606**		
	**N**	**%**	**N**	**%**		**N**	**%**	
**Age distribution** (years)					**<0.001**			**<0.001**
20 – 34	6 630	23.7	3 113	14.3		494	3.4	
35 – 49	8 671	31.0	6 132	28.1		3 967	27.2	
50 – 64	6 589	23.5	7 352	33.7		5 910	40.5	
65 +	6 101	21.8	5 207	23.9		4 235	29.0	
**BMI**					**<0.001**			**<0.001**
Underweight (≤ 18.4)	267	1.0	182	0.8		89	0.6	
Normal (18.5 – 24.9)	12 169	43.8	8 212	37.8		5 095	35.0	
Overweight (25.0 – 29.9)	10 301	37.1	8 260	38.0		5 839	40.1	
Obese (≥ 30)	5 028	18.1	5 072	23.3		3 538	24.3	
**Self-perceived health status**					**0.038**			**<0.001**
Bad	449	1.6	274	1.3		176	1.2	
Not entirely good	7 312	26.3	5 569	26.4		4 007	28.4	
Good	15 488	55.8	11 834	56.1		7 935	56.2	
Excellent	4 505	16.2	3 424	16.2		2 006	14.2	
**Smoking**					**0.095**			**0.009**
Not smoking	19 651	71.2	15 273	71.4		10 395	73.1	
Smoking	7 952	28.8	5 975	28.1		3 818	26.9	
**Glasses alcohol last 2 weeks**					**<0.001**			**0.016**
0	7 002	34.0	5 947	32.3		3 805	30.9	
1 – 4	11 425	55.5	9 421	51.2		6 356	51.6	
>5	2 160	10.5	3 047	16.5		2 147	17.4	
**Parity**					**<0.001**			**<0.001**
0	3 931	14.2	2 633	12.1		895	6.1	
1	3 229	11.7	2 090	9.6		1 150	7.9	
2	8 951	32.3	7 633	35.1		5 446	37.3	
3+	11 595	41.9	9 415	43.2		7 101	48.7	
**Hysterectomy**	1 773	13.4	2 040	10.6	**<0.001**	1 574	11.2	**0.111**
**Asthma**	2 390	8.6	2 318	10.6	**<0.001**	1 534	10.5	**0.694**
**Chronic cough**	829	3.0	881	4.1	**<0.001**	605	4.2	**0.615**
**Diabetes**	739	2.6	816	3.7	**<0.001**	613	4.2	**0.029**
**Angina**	979	3.5	534	2.4	**<0.001**	415	2.8	**0.021**
**Heart attack**	415	1.5	327	1.5	**0.893**	258	1.8	**0.047**
**Stroke**	459	1.6	474	2.2	**<0.001**	368	2.5	**0.032**

^a^: Women with a valid answer to the entry question in EPINCONT.

^b^: p - values from chi - square tests comparing the women who participated in E1 with those who participated in E2.

^c^: Variables from HUNT3 were used.

^d^: p - values from chi - square tests comparing women who participated in E2 with those who participated in both E1 and E2.

**Table 3:** Prevalence of any UI, type and severity of UI in EPINCONT1 and EPINCONT2 in 5 – year age groups

**Age - group**	**Survey**	**Any UI**		**p**	**Type of UI**					**P**	**Severity of UI**					**p**
					**N**	**Stress**	**Urgency**	**Mixed**	**Other**		**N**	**Slight**	**Moderate**	**Severe**	**Very Severe**	
		**N**	**%**			**%**	**%**	**%**	**%**			**%**	**%**	**%**	**%**	
**20 - 24**	**E1**	222	11.2	**0.951**	218	43.1	12.4	30.3	14.2	**0.326**	172	53.5	36.6	8.1	1.7*	**0.220**
	**E2**	116	11.3		116	46.6	18.1	24.1	11.2		113	63.7	30.1	6.2	0*	
**25 - 29**	**E1**	320	15.0	**0.001**	318	54.4	11.9	26.4	7.2	**0.139**	229	55.5	39.3	3.9	1.3*	**0.310**
	**E2**	173	20.0		173	54.9	17.9	19.1	8.1		170	61.8	35.3	2.9	0*	
**30 - 34**	**E1**	479	19.1	**<0.001**	479	57.6	9.8	26.7	5.8	**0.595**	343	59.5	38.2	1.7	0.6*	**0.756**
	**E2**	299	24.5		299	53.2	12.0	29.1	5.7		297	63.3	35.0	1.3	0.3*	
**35 - 39**	**E1**	576	21.0	**<0.001**	570	59.5	6.8	28.8	4.9	**0.571**	432	57.6	39.6	2.5	0.2*	**0.640**
	**E2**	528	29.3		525	58.1	9.1	28.2	4.6		516	57.9	38.8	3.3	0*	
**40 - 44**	**E1**	728	24.6	**<0.001**	724	61.0	8.3	27.3	3.3	**0.230**	511	51.5	42.7	5.1	0.8*	**0.031**
	**E2**	633	30.5		633	56.9	10.4	30.2	2.5		624	53.7	43.9	2.1	0.3*	
**45 - 49**	**E1**	851	28.7	**0.098**	849	63.3	6.5	27.7	2.6	**<0.001**	594	52.2	39.7	6.6	1.5	**0.166**
	**E2**	695	30.8		691	49.8	11.3	36.2	2.7		681	47.9	45.7	5.4	1.0	
**50 - 54**	**E1**	832	30.3	**0.039**	828	54.2	7.6	36.0	2.2	**0.034**	554	45.7	43.0	10.5	0.9*	**0.039**
	**E2**	663	27.6		660	47.1	10.5	40.0	2.4		645	46.2	47.0	6.0	0.8*	
**55 - 59**	**E1**	567	28.1	**0.371**	558	50.9	9.7	36.4	3.0	**0.023**	394	36.5	49.7	11.7	2.0	**0.032**
	**E2**	725	29.4		721	43.7	14.0	38.3	4.0		694	44.5	46.0	8.4	1.2	
**60 - 64**	**E1**	480	26.3	**0.008**	474	40.9	10.5	45.4	3.2	**0.207**	297	35.4	45.5	16.5	2.7	**0.091**
	**E2**	746	30.0		741	38.6	14.7	44.0	2.7		720	35.8	51.0	11.1	2.1	
**65 - 69**	**E1**	513	27.9	**0.240**	500	36.0	14.6	44.8	4.6	**0.009**	312	22.4	51.6	20.8	5.1	**0.052**
	**E2**	529	29.6		526	30.0	16.7	51.3	1.9		509	29.5	51.1	16.3	3.1	
**70 - 74**	**E1**	572	31.6	**0.302**	563	32.9	15.5	46.4	5.3	**0.159**	390	21.5	50.8	22.6	5.1	**0.307**
	**E2**	458	33.4		456	27.4	18.4	50.0	4.2		438	23.5	52.3	21.5	2.7	
**75 - 79**	**E1**	481	34.9	**0.864**	474	32.9	19.2	40.9	7.0	**0.024**	317	22.1	44.5	25.9	7.6	**0.920**
	**E2**	367	34.6		362	25.7	17.1	51.4	5.8		342	21.1	45.9	26.6	6.4	
**80 +**	**E1**	386	36.0	**0.037**	366	29.8	19.7	41.0	9.6	**0.197**	238	14.7	42.9	35.7	6.7	**0.713**
	**E2**	400	40.4		389	23.7	20.6	47.3	8.5		370	16.5	44.6	31.4	7.6	
**Total**	**E1**	7 008	25.0	**<0.001**	6921	49.4	10.9	35.0	4.7	**<0.001**	4 784	41.9	43.5	12.1	2.5	**0.006**
	**E2**	6 332	29.0		6292	42.9	13.9	39.3	4.0		6119	42.1	45.5	10.5	1.9	

*: ≤ 5 participants.

**Table 4:** Incidence and remission of any UI and distributions by type and severity in EPINCONT

	**Any UI**		**Type of UI**								**Severity of UI**							
			**Stress**		**Urgency**		**Mixed**		**Other**		**Slight**		**Moderate**		**Severe**		**Very Severe**	
	**N**	**%**	**N**	**%**	**N**	**%**	**N**	**%**	**N**	**%**	**N**	**%**	**N**	**%**	**N**	**%**	**N**	**%**
**Incidence of UI**																		
**Age / years**^**a**^																		
20 – 39	897	22.3	493	55.1	108	12.1	262	29.3	32	3.6	536	61.1	322	36.7	16	1.8	3	0.3
40 – 54	665	15.9	300	45.4	105	15.9	228	34.5	28	4.2	323	50.1	296	45.9	23	3.6	3	0.5
55 +	492	17.9	157	32.4	93	19.2	201	41.5	33	6.8	142	31.0	222	48.5	80	17.5	14	3.1
*Total*	2 054	18.7	950	46.6	306	15.0	691	33.9	93	4.6	1 001	50.6	840	42.4	119	6.0	20	1.0
**Remission of UI**																		
**Age / years**^**a**^																		
20 – 39	322	37.2	178	56.2	33	10.4	73	23.0	33	10.4	152	64.7	76	32.3	6	2.6	1	0.4
40 – 54	620	36.8	384	62.1	48	7.8	161	26.1	25	4.0	258	60.3	153	35.7	17	4.0	0	0
55 +	296	27.4	156	54.0	26	9.0	91	31.5	16	5.5	93	46.3	87	43.3	16	8.0	5	2.5
*Total*	1 238	34.1	718	58.7	107	8.7	325	26.6	74	6.0	503	58.2	316	36.6	39	4.5	6	0.7

^a^: Age when participating in EPINCONT1 (E1).

**Table 5:** **Adjusted**^**a **^**Odds Ratios (OR) for incidence and remission of UI in women participating in both EPINCONT1 (E1) and EPINCONT2 (E2)**

		**Incidence of UI**				**Remission of UI**		
	**N = 2 054**	**OR**	**95% CI**		**N = 1 238**	**OR**	**95% CI**	
**Age** (E1)	2 054	0.99	0.99 – 1.00	***	1 238	0.98	0.98 – 0.99	***
**BMI** (E1)	2 046	1.03	1.02 – 1.04	***	1 233	0.96	0.95 – 0.98	***
**Weight Change** (kg)								
No change (-3 / +3)	651	1.0			475	1.0		
Loss: 3 - 10	209	1.01	0.85 – 1.20		168	1.07	0.85 – 1.34	
Loss: > 10	70	1.28	0.96 – 1.72		52	1.33	0.91 – 1.93	
Gain: 3 - 10	720	1.29	1.14 – 1.45	***	395	0.90	0.76 – 1.06	
Gain: > 10	377	1.71	1.47 – 1.99	***	130	0.69	0.54 – 0.88	**
**Parity** (E2)								
0	111	1.0			50	1.0		
1	166	1.37	1.04 – 1.79	*	89	0.90	0.58 – 1.40	
2	736	1.28	1.03 – 1.61	*	450	0.97	0.67 – 1.40	
3 +	1 040	1.56	1.26 – 1.95	***	646	0.96	0.67 – 1.38	
**Smoking** (E1)	541	0.93	0.83 – 1.04		337	1.09	0.92 – 1.28	

^a^: All OR are adjusted for all the other variables presented in the table.

*: p < 0.05. **: p < 0.01. ***: p < 0.001.

A new category was added to the smoking variable in HUNT3 compared with HUNT2; -smoking occasionally. Those who reported smoking occasionally were included as current smokers in all analyses.

### Ethics

The main study had ethical recommendation from both the Regional and National Ethics Review Boards. Attendance was completely voluntary and subjects gave and extensive written consent to use of the collected material. The survey was also approved by the Norwegian Data Inspectorate.

### Statistics

Data were analysed using PASW Statistics version 18.0. Statistical significance was accepted at a 5% level (p < 0.05). Age and BMI of the participants in E1 and E2 were presented as means with Standard Deviations (SD).

Chi - square tests were used to compare characteristics of the women who had participated in E1, E2 or both. Mean age and BMI in E1 and E2 were compared with t – tests.

Logistic regression analyses were performed with incidence or remission as dependent variables. Women who were continent and women who were incontinent on both occasions were used as reference categories for incidence and remission of UI respectively. Among the adjustment variables age and BMI were used as continuous variables. The rest of the adjustment variables were treated as categorical and the following reference values were used: nulliparous women, weight change between -3 /+3 kilos and no smoking. Odds ratios (ORs) with corresponding 95% confidence intervals (CI) were the effect measures.

## Results and discussion

### Results

Table 1 displays participation rates for the two HUNT surveys as well as the EPINCONT parts, E1 (1995-1997) and E2 (2006-2008). Most women who attended the screening stations and received Q2 (containing the EPINCONT questions) chose to participate and thus response rates were close to 80% in both EPINCONT surveys.

Approximately 11 years after the E1 survey the crude UI prevalence had increased significantly from 25.0% (N = 7 008) in E1 to 29.0% (N = 6 332) in E2 (p < 0.001). The women who participated in E2 were older, with a mean age of 53.0 years (± 15.9 SD) compared with 49.3 years (± 17.1 SD) in E1 (p < 0.001). The mean age of the women participating in both surveys was 57.6 (± 13.2 SD) years of age. A rise in mean BMI was found, from 26.21 (± 4.54 SD) in E1 to 26.96 (± 4.84 SD) in E2 (p < 0.001). The mean BMI for women who attended both studies was 27.19 (± 4.73 SD).

**Figure 1:**
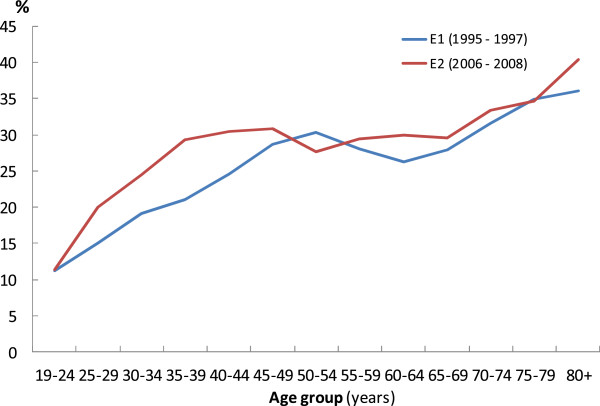
Prevalence (%) of UI in EPINCONT1 (E1) and EPINCONT2 (E2) by 5-year age groups.

Figure 1 shows crude UI prevalence in 5-year age groups. In E1 a peak was observed in the group 50 – 54 years of age, the peak was followed by a decline and around 62 years of age the prevalence started to increase again. A similar pattern was observed among participants in E2, but the overall prevalence was higher, the peak occurred at a younger age, and the decline in E2 came at the age of the peak in E1.

Table 2 displays characteristics of the women participating in E1, E2 as well as the women who participated in both surveys. The women who participated in E2 were significantly older, had a higher BMI as well as more overweight and obesity, and they consumed more alcohol compared with the women in E1. Other statistically significant differences were more asthma, chronic cough, diabetes and stroke in the participants in E2, they also had less angina, fewer hysterectomies, while nulliparous women were more common in E1. The women who participated in both EPINCONT surveys, had even more overweight and obesity as measured by BMI, they smoked less and consumed more alcohol compared with all the E2 women. They also had a higher prevalence of angina, heart attack, stroke and diabetes compared with all the E2 women, while fewer of them were nulliparous.

Prevalence of any UI, type of UI and severity of UI in 5 - year age groups are presented in Table 3 for the E1 and E2 women. The women in E2 had higher prevalence of UI in all age groups except for the groups 50 – 54 and 75 - 79 years of age (see also Figure 1). The differences in prevalence of any UI were statistically significant in most age groups. Stress UI was overall the most prevalent type of UI in both E1 and E2, followed by mixed, urgency and other UI. Mixed UI became the most prevalent type of UI after 60 years of age in both E1 and E2. The type distribution among E1 and E2 women was relatively similar, but was significantly different in the age groups 45 - 59, 65 - 69 and 75 - 79 years, with less stress UI among E2 women compared with the E1 women. Moderate severity dominated in both E1 and E2, followed by slight, severe and very severe UI. Slight UI was most prevalent in the younger age groups, and moderate UI was the most prevalent grade of severity after 50 years of age in E2 and after 55 years in E1. In three age groups (40 – 44, 50 – 54 and 55 – 59 years of age) there were significant differences in severity distribution in E1 compared with E2, with less severe symptoms in E2.

Table 4 shows incidence and remission of UI by type and severity for the women participating in both EPINCONT surveys (E1 and E2), divided into three age groups (using the age on participation in E1). A total of 2 054 (18.7%) women reported incident UI whilst 1 238 (34.1%) reported remission of UI. The average yearly incidence rate was 1.7%. Incidence of UI was highest in the youngest women (20 - 39 years (E1)). Most of the women with incident UI experienced stress UI (46.6%), followed by mixed (33.9%), urgency (15.0%) and other UI (4.6%). After 55 years of age (E1) incident UI was mostly of mixed type. Slight severity was the most common grade of severity, with 50.6% of the women with incident UI reporting this severity degree. After 55 years of age (E1), moderate UI was the most prevalent grade of severity, 48.5%. The average yearly remission rate was 3.1%. Remission was highest in women younger than 55 (E1), and most women who experienced remission had stress type and slight severity at baseline in E1. This was the case for all age groups.

Table 5 displays adjusted odds ratios for incidence and remission of UI. Parity and increasing BMI were found to be associated with increased odds of incident UI, whilst increasing age reduced the odds of incident UI, with a 1% reduction in incident UI for each year increase in age. Weight gain was also associated with more incidence of UI, and a certain dose response effect was found. Increasing age and BMI, as well as large weight gains (≥ 10 kilos) were associated with reduced odds for remission of UI.

Separate logistic regression analyses were performed to investigate diabetes as a risk factor for incidence and remission of UI. Having diabetes in E1 was not found to be significantly associated with neither incidence nor remission of UI. However, getting diabetes between E1 and E2 was found to reduce the odds of remission significantly before adjustments (OR 0.68, 95% CI: 0.48 – 0.96). The association was not significant after adjustments for age, BMI, weight change, parity and smoking (OR 0.74, 95% CI: 0.51 – 1.07).

### Discussion

In this follow-up study of the large Norwegian E1 survey we found a 16% relative increase in crude UI prevalence, from 25.0% to 29.0% in approximately 11 years. The incidence and remission of UI among those participating in both surveys were found to be 18.7% and 34.1% respectively. Both incidence and remission were found to be highest in women 20 – 39 years of age. Adjusted logistic regression analyses identified known risk factors such as BMI, weight change and parity to be influential on the odds ratios for incidence and remission of UI. Increase in age was found to be associated with reduced incidence and remission of UI, whilst increase in BMI increased the odds of incident UI and reduced the odds of remission. Increasing weight gain increased the incidence of UI, with a dose response effect. Weight gains above 10 kilos reduced the odds of remission.

One of the strengths of the EPINCONT study is the substantial number of women (14 606) participating in both surveys. The study is population based, and all adult women 20 years of age and older were invited to participate. This makes it reliable to transfer knowledge back to the entire adult female population. In addition the EPINCONT study is part of a larger health study (HUNT) which provides extensive information about other health topics in these women, which could be helpful to understand changes in UI status. EPINCONT uses definitions of UI as recommended by the ICS [14] and a validated Severity index [13].

The surveys have some limitations. The definition chosen for any UI in EPINCONT is in accordance with ICS guidelines. This is a broad definition, and hence women with clinically unimportant UI would also be identified and included in our analyses. Investigating the level of bother in the women with incident UI, we found that 70% of the women with incident UI experienced no bother or only a small nuisance due to their UI. Selection bias could also be a limitation to our study, due to the decline in participation in HUNT3. Of the invited women 58.5% participated in HUNT3. However, the response rate to the EPINCONT part of the study remained quite high (78.7%) compared with a response rate of 81.0% in E1. Drop out of women before they received Q2 could induce falsely lower prevalence and incidence as well as a higher remission rate of UI, since healthier people tend to participate to a higher degree in studies like these. Using our data to compare women who only participated in E1 with those who participated twice, the women who participated only in E1 smoked more and were younger (mean age 43.3 years compared with 46.4 years). In addition there were only small and non-significant differences with regard to diseases such as diabetes, asthma and myocardial infarction. The prevalence of UI was lower in the group of women participating only in E1 (22.3% compared with 24.9% in those participating twice. Details are not shown). This indicates that UI prevalence is not underestimated despite a lower attendance in HUNT3. Another limitation is the 11 year period between the two studies. Other incidence and remission studies which have been conducted over a shorter time span have found quite high incidence and remission rates [17,18] indicating that UI is a fluctuating condition that comes and goes. Thus, the long time passed between the two surveys, and only two points of data collection, makes it difficult to discover short time changes in UI status, type and severity, and could partly explain the low incidence and remission rates found.

The prevalence of UI found in the two EPINCONT surveys within the range of reported prevalence in other longitudinal studies, where prevalence’s between 15 [2] - 66% [17] have been found. Some of this span could be due to different age groups included in the studies. Prevalence curves from the two EPINCONT surveys show approximately the same pattern with the highest prevalence of UI in the older age groups. A similar prevalence pattern was found in another longitudinal cohort study from Norway [19]. Since the EPINCONT surveys include women 20 years or older a lower prevalence of UI is expected compared with studies conducted on older women [8,18]. The majority of women in both E1 and E2 were found to have stress UI of moderate severity. Stress UI is known to be the most prevalent type of UI in young and middle-aged women, whilst urgency and mixed UI increases with age [15,20]. Since most women in our surveys were below 60 years of age, stress UI was expected to be the most prevalent type. This type distribution is also in accordance with other studies [19,21], and compared with another Norwegian longitudinal study [22], the EPINCONT women had the same type and severity distribution for the equivalent age group, though more of the EPINCONT women reported a new onset UI of severe degree.

During the eleven years between the two EPINCONT surveys we found a 16% relative increase in crude UI prevalence from 25.0% to 29.0. Repeated cross – sectional studies including entire adult female populations (20 +) is the only way to investigate populational changes in UI prevalence, and to our knowledge the National Health and Nutrition Examination Survey (NHANES study) is the only study who have published changes in UI prevalence in a representative sample of the adult female population [5]. The change found in crude UI prevalence in EPINCONT is in accordance with the findings from the NHANES study, with relative increases of 16.0% in 11 years and 7.9% in 6 years, respectively [5]. The prevalence increase in EPINCONT could either represent an actual increase in UI prevalence in adult females, be due to increase in known risk factors for UI, or be caused by to the lower attendance in HUNT3, making selection bias more likely to occur. Known risk factors for UI such as age [23,24], BMI [25] and parity [26] were found to be significantly different in E1 and E2, and the low participation rate in HUNT3 could represent a potential selection bias if the women who had UI were more prone to participate in the E2 study. The UI prevalence was as previously mentioned higher in the E1 women who also participated in E2 (24.9%) compared with the prevalence in the E1 women who just participated in E1 (22.8%). However, the prevalence in the women participating twice were closer to the total prevalence in HUNT2 (25.0%), and given the good response rate in HUNT2 selection bias is less likely to be a major determinant of increased prevalence.

An average yearly incidence rate of 1.7% puts the EPINCONT study in the lower range compared with incidence reported in other longitudinal studies [7,27,28]. Not many studies exist which have been conducted on a similar population and have used the same UI definition: defining an incident case of UI as someone who reports no leakage at baseline and “any leakage” regardless of amount and frequency at follow-up. In one similar study conducted in Gothenburg on women older than 20 randomly selected from the Swedish National population register, an incidence rate of 21% in 16 years was found, providing an average yearly incidence of 1.3% [2]. That study also found the highest incidence of UI to occur in the youngest women (20 - 34 years) which is similar to our study. A study on women 20 – 89 years of age conducted in Austria showed a yearly incidence of any UI of 3.9% [3]. The incidence found in the EPINCONT women is quite similar to that found in Sweden for the same age group, with only two data points separated by a long time span. Many of the other longitudinal studies on UI have included a limited age span [4,10,28] or restricted the definition of “*any UI*” to apply only if the participants reported a certain frequency of leakage [3,7,10,18]. Such differences in age and UI definitions might partly explain the wide range in incidences from 0.9% [29] per year to 19% [17]. The EPINCONT women who became incontinent between studies primarily got stress UI of slight severity. In women older than 55 mixed UI of moderate severity was more common. This type and severity distributions for new onset UI were similar to the ones found in another longitudinal cohort study from Norway [22]. In the large Nurses’ Health Study two year incidence of frequent UI was also primarily stress UI, followed by mixed and other UI [7]. And in another publication from the same cohort an increased risk of severe UI in women older than 60 years, as well as an increased risk for urgency and mixed type of UI was reported [30], which corresponds well with our findings.

The average yearly remission rate of 3.1% in the EPINCONT women also falls into the lower range compared with existing literature, where reported yearly remission rates lies between 2.1% [2] and 27.8% [10]. Another similar study found a remission rate of 2.9% [3], though defining UI as any involuntary leakage during the last month. Remission rate in white women in the Nurses’ Health Study was a little higher, 4.5%, but did not include women younger than 37 years of age [31].

Other studies have identified age [32], BMI [25,33], weight gain [25,33], oral contraceptive use [34] and physical function and psychiatric illness [28,35] among risk factors associated with incident UI. In the current study we identified BMI as a risk factor for incident UI, whilst weight change and parity were found to be associated with incident UI. Weight change has been found to be influential on UI status, and weight loss programs have proven to be an effective way to improve UI and increase remission of UI [25,36,37]. There was an association between weight gain above 10 kilos and incidence and remission of UI in the EPINCONT women, where incidence of UI was increased and remission of UI was reduced with a weight gain of 10 kilos or more. However, we found no significant association between UI (neither incidence nor remission of UI) and weight loss between the two EPINCONT surveys. This could be due to few participants in the largest weight loss group (lack of power) or other factors difficult to control in cross - sectional studies (such as loss of weight associated with serious disease). In previous publications from the E1 survey we found diabetes to be significantly associated with prevalent UI [38,39]. Having diabetes in E1 was not found to be associated with incidence or remission of UI in neither bivariate nor multivariate analyses. This is in accordance with findings by Waetjen et al, who found diabetes to be associated with prevalence of any UI (OR 2.34, 95% CI: 1.21 – 4.55) but not incidence of any UI [28]. Jackson et al found peripheral diabetes neuropathy to be borderline predictive of any incident UI (OR 1.7, 95% CI: 1.0 – 3.1) [17]. We did however find an association between recently developed diabetes (after E1) and remission of UI, where development of diabetes between E1 and E2 was associated with reduced odds of remission from UI. This could be due to the different type and severity distribution of UI found in women with diabetes [38], where women with diabetes were found to have a higher prevalence of mixed and severe UI, or caused by higher prevalence of other risk factors for UI in women with diabetes (such as overweight). The results might indicate an overrepresentation of other risk factors for UI in women with diabetes, since the association between recently developed diabetes and remission of UI no longer was significant after adjustments for age, BMI, weight change, parity and smoking. The lack of any significant results in the analyses of having diabetes in E1 as a risk factor for incidence or remission of UI could be due to the limited number of diabetes patients participating in both EPINCONT surveys (n = 176, of whom 64 reported any UI in E2).

## Conclusion

A 16% relative increase in crude UI prevalence was found in EPINCONT, and average yearly incidence and remission rates were 1.7% and 3.1% respectively. Known risk factors for UI such as age, BMI, weight change and parity were found to be influential on incidence of UI, remission of UI or both. Getting diabetes between the two EPINCONT studies reduced the odds of remission of UI, though only before adjustments. Some of the increase in UI prevalence in EPINCONT could be explained by concurrent changes in risk factors in the study population.

## Abbreviations

BMI: Body Mass Index; UI: Urinary incontinence; EPINCONT: EPidemiology of INcontinence in the County of Nord-Trøndelag; E1: EPINCONT1; E2: EPINCONT2; HUNT: The Nord-Trøndelag Health study; OR: Odds ratios; CI: Confidence interval; SD: Standard deviation.

## Competing interests

The authors declare that they have no competing interests’.

## Authors’ contributions

All authors designed and planned the study. MHE prepared the file for analyses and conducted the analyses. MHE drafted the first manuscript, whilst all authors contributed to the final manuscript. All authors approved of the final manuscript.

## Pre-publication history

The pre-publication history for this paper can be accessed here:

http://www.biomedcentral.com/1471-2490/13/27/prepub
